# Progress with PfSPZ Vaccine, a radiation attenuated *Plasmodium falciparum* sporozoite vaccine

**DOI:** 10.1186/1475-2875-13-S1-O34

**Published:** 2014-09-22

**Authors:** Peter Billingsley, B Kim Lee Sim, Eric James, Thomas Richie, Seif Shekalaghe, Sara Healy, Mahamadou Sissoko, Benjamin Mordmueller, Julie Ledgerwood, Barney Graham, Patrick Duffy, Robert Seder, Kirsten Lyke, Judith Epstein, Pedro Alonso, Salim Abdullah, Ogobara Duombo, Peter Kremsner, Marcel Tanner, Stephen Hoffman

**Affiliations:** 1 Sanaria Inc., Rockville, MD, USA; 2 Ifakara Health Institute, Bagamoyo, Tanzania; 3 Laboratory for Malaria Immunology and Vaccinology, NIAID-NIH, Bethesda, MD, USA; 4 Malaria Research and Training Cente, Bamako, Mali; 5 Institut für Tropenmedizin, Tübingen, Germany; 6 Vaccine Research Center, NIAID-NIH, Bethesda, MD, USA; 7 Center for Vaccine Development, University of Maryland, Baltimore, MD, USA; 8 Navy Medical Research Center, Bethesda, MD, USA; 9 Barcelona Centre for International Health Research, Barcelona, Spain; 10 Swiss Tropical and Public Health Institute, Basel, Switzerland

## 

Sanaria^®^ PfSPZ Vaccine is composed of aseptic, purified, cryopreserved, attenuated (non-replicating), metabolically active *Plasmodium falciparum* (Pf ) sporozoites (SPZ) produced in compliance with good manufacturing practices (GMPs) that meet all regulatory standards. This vaccine provided full protection against Pf infection in 100% (6/6) volunteers, who received five doses of 1.35 × 10^5^ PfSPZ administered intravenously in a study at the Vaccine Research Center (VRC), NIAID, NIH [1]. Based on these data, the PfSPZ Vaccine Clinical Consortium composed of investigators from USA, Africa, and Europe has developed a four stage clinical development plan (CDP) that maps out a 4-5 year timeline to licensure and a large scale demonstration project to eliminate malaria from an island population in Africa. In 2014, six different clinical trials of PfSPZ Vaccine at seven clinical sites in the United States (Bethesda, Baltimore, Silver Spring), Mali, Tanzania, Equatorial Guinea, and Germany will be underway. These six clinical trials, which include >450 subjects, comprise Stage 1 of the four stage PfSPZ Vaccine CDP. They are designed to 1) assess the reproducibility of the data generated in the VRC study and 2) assess and optimize durability of protection, protection against heterologous strains of Pf, reduction in numbers of doses, immune assays that predict protection, implementation of immunization, and alternative route of administration. We will provide an update of these stage 1 clinical trials and plans for stage 2 studies that will address questions required for progressing to pivotal phase 3 clinical trials in stage 3, and to demonstration projects for focal elimination in small populations.
